# Ventral striatal activity links adversity and reward processing in children

**DOI:** 10.1016/j.dcn.2017.04.002

**Published:** 2017-04-15

**Authors:** Niki H. Kamkar, Daniel J. Lewis, Wouter van den Bos, J. Bruce Morton

**Affiliations:** a Department of Psychology, University of Western Ontario, 361 Windermere Road, Westminster Hall, London, Ontario N6A 3K7, Canada; b Center for Adaptive Rationality (ARC), Max-Planck-Institute for Human Development, Berlin 14195, Germany; c The Brain and Mind Institute, Natural Sciences Centre, Room 120, Western University London, Ontario, Canada, N6A 5B7

**Keywords:** Delay discounting, Early-life adversity, fMRI, Impulsivity, Ventral striatum

## Abstract

Adversity impacts many aspects of psychological and physical development including reward-based learning and decision-making. Mechanisms relating adversity and reward processing in children, however, remain unclear. Here, we show that adversity is associated with potentiated learning from positive outcomes and impulsive decision-making, but unrelated to learning from negative outcomes. We then show via functional magnetic resonance imaging that the link between adversity and reward processing is partially mediated by differences in ventral striatal response to rewards. The findings suggest that early-life adversity is associated with alterations in the brain’s sensitivity to rewards accounting, in part, for the link between adversity and altered reward processing in children.

Individual differences in reward-based learning and decision-making take root early in life and predict a host of later outcomes including physical and psychological health, financial well-being, academic achievement, and social adjustment (Mischel et al., 1989, Moffitt et al., 2011, Schlam et al., 2013, Shoda et al., 1990). Understanding the origins of early differences in reward processing is therefore an important research goal.

The current study focused on potential links between reward-based learning and decision-making early in development and exposure to adverse life events. In humans, adversity has been linked to a variety of alterations in reward processing, including both potentiated and attenuated motivation to approach prospective rewards. For example, adversity experienced by traumatized children is associated with heightened impulsivity and hyperactivity (Laucht et al., 2007), increased risk of substance use and addiction (Sinha, 2008), riskier sexual behavior (Cinq-Mars et al., 2004, Noll et al., 2009, Senn et al., 2007), and heightened incidence of obesity (Davis et al., 2014, Farr et al., 2015, Non et al., 2016), but also anhedonia and depression (Bogdan and Pizzagalli, 2006, Pizzagalli et al., 2007). From the use of animal models, it is known that adult rodents who, as pups, were separated from their mother show greater impulsivity, sensitivity to rewards, and behavioral inflexibility compared to adults who were reared naturally (Hall et al., 1998, Lovic et al., 2011). And in rhesus monkeys, individuals at the bottom of a social hierarchy show greater self-administration of cocaine compared to individuals at the apex of the hierarchy (Morgan et al., 2002). Why − on a very basic level − adversity is associated with alterations in reward processing, and whether this association extends to non-traumatized children remains unclear.

Our hypothesis is that variation in the quality of early experience will have implications for the functional calibration of the brain’s reward system later in childhood. The reward system is an evolutionarily well-conserved network of subcortical and cortical brain regions that includes the ventral tegmental area (VTA), the ventral striatum (VS), ventromedial and orbital prefrontal cortex (vmPFC, OFC), and dorsal anterior cingulate cortex (dACC). Although functionally multifaceted, these regions all utilize dopaminergic signals to support different aspects of reward-based learning and decision-making. According to developmental and evolutionary theory (Gatzke-Kopp; Gluckman and Hanson, 2004, Meaney, 2001, Meaney, 2010), biological systems, such as the reward system, enjoy considerable functional plasticity early in development, making them highly attuned to indicators of environmental quality. Thus, adjustments in dopamine-related phenotypic traits − including dopamine availability, dopamine receptor density, and dopamine-mediated behavioral traits such as learning rate to rewards, activity level, and novelty seeking − can occur in response to variation in environmental quality, including variation that falls within a normative range (Gatzke-Kopp, 2011, Gluckman and Hanson, 2004). These adjustments may be adaptive in the short-term, but carry the burden of increased risk of adverse outcomes such as addiction and psychopathology. As such, adverse early-life experiences can become “biologically embedded” in the developing brain (Hertzman, 1999, Hertzman, 2012) and exert a lasting influence on the physical and psychological health of the affected individual (Brake et al., 2004, Hall et al., 1999, Hall et al., 1998, Léonhardt et al., 2007, Nelson, 2013).

Accumulating evidence, particularly from the use of animal models, is consistent with the idea that adversity impacts reward-based learning and decision-making via its influence on dopamine signaling in the reward system (Abercrombie et al., 1989, Hall et al., 1998, Piazza and Le Moal, 1996, Lovic et al., 2011). In rodent and primate models, for example, adversity has been associated with elevated levels of basal dopamine (DA) in the ventral striatum (VS), potentiated dopamine response to amphetamine administration in the VS (Piazza and Le Moal, 1996; Hall et al., 1996), lower D2 receptor density in the VS (Hall et al., 1998, Morgan et al., 2002), and potentiated responses in dopamine-mediated traits such as novelty-seeking and activity level (Lovic et al., 2011, Piazza and Le Moal, 1996). These effects appear to extend to humans, as early-life adversity has been associated with elevated levels of striatal dopamine (Egerton et al., 2016; Preussner et al., 2004), and increased striatal dopamine release to rewarding stimuli such as amphetamine (Oswald et al., 2014). Whether these associations hold for normative levels of adversity − as might be predicted (Gatzke-Kopp, 2011, Gluckman and Hanson, 2004) − is unknown.

The goals of the present study were therefore twofold. The first goal was to examine whether normative variation in exposure to adverse early-life events is associated with variation in children’s reward-based learning and decision-making. To test this question, we recruited a sample of typically-developing children and assessed exposure to a variety of events that while adverse, would be considered within the range of normative experience. We then assessed reward-based learning and decision-making using tasks that are dependent, at least in part, on dopamine signaling in the reward system. The first was the Probabilistic Selection Task (PST), a measure of reward-based approach-avoidance learning that has been shown, via genetics methods and the study of Parkinson’s patients, to be sensitive to variation in levels of striatal dopamine (Frank et al., 2004). The second was a Delay Discounting (DD) task, a measure of inter-temporal (or impulsive) choice which has been shown to be associated with striatal reactivity to the provision of rewards (Hariri et al., 2006). Having tested for an association between adversity and reward-based learning and decision-making, the second goal of the study was to examine whether possible associations between adversity and reward processing were explainable at least in part, by the functional response of the reward system to small gains and losses. To test for this possibility, children were administered a reinforcement learning task as brain activity was measured via functional magnetic resonance imaging (fMRI). The task provides a means of measuring behavioral and brain response to both gains and losses separately (Pessiglione et al., 2006). Importantly for the goals of the current study, learning rates and ventral striatal response − specifically to gains − covary with pharmacologically-induced changes in striatal dopamine, with these effects measureable using fMRI (Pessiglione et al., 2006). Use of this task in combination with fMRI, then, provided a validated, albeit indirect measure of reward-related dopaminergic transmission.

Our predictions were as follows:

• Greater experience of early-life adversity within the normative range would be associated with potentiated reward-learning and more impulsive decision-making.
• The association between adversity and reward-based learning and decision-making would be explainable, at least in part, by differences in the functional response of regions within the reward system to the receipt of small gains.

## Method

Two studies examined the association between normative variation in early-life adversity and children’s reward-based learning and decision-making. The first focused on associations between adversity and reward-related behaviors; the second focused on neurophysiological mediators of adversity and reward-behavior associations.

## **1** Method study 1

### **1.1** Participants

Trained research assistants recruited 40 (24 females) children between the ages of 9–12 years (M = 10.75, SD = 0.95) from a database of London, Ontario families who voluntarily participate in psychological research. Children who had experienced “trauma” (e.g., physical/sexual abuse, witnessing death or severe injury of a family member/close friend) or had any developmental, neurological, or psychiatric disorders were excluded from the study. Parents provided written consent to their children’s participation; children provided verbal assent. Child participants received a $25 gift card for participating. Parents were compensated for travel and parking expenses. All aspects of the study were conducted in accordance with the Declaration of Helsinki.

### **1.2** Measure of early-life adversity

To measure normative variation in exposure to adverse early-life events, parents of child participants were administered the Early Life Experiences Questionnaire (ELEQ). The measure consists of a list of 22 different adverse events (e.g., changing schools, moving to a new neighborhood, loss of a pet, loss of a grandparent) that would all be emotionally challenging for a child, but within the range of normative experience. For each event, parents indicated how frequently and how intensely their child had experienced the event. Parents could also add frequency and intensity estimates for additional events that were not included in list of 22-events. A score for individual events was computed as the product of frequency and intensity, and a Total Adversity score was calculated for each participant as the sum of all individual event scores.

Use of the ELEQ was motivated by our interest in normative variation in adversity exposure. Other instruments, such as The Juvenile Victimization Questionnaire, or The Adverse Childhood Experiences Questionnaire, while valuable measures, profile exposure to more severe and non-normative forms of adversity including maltreatment, physical abuse, sexual abuse, warfare, violence, and victimization (Felitti et al., 1998, Finkelhor et al., 2005).

### **1.3** Measures of reward-based learning and decision-making

Reward-based learning was assessed by means of the Probabilistic Selection Task (PST), a measure of reinforcement learning which has previously been shown to be sensitive to variation in levels of striatal dopamine (Frank et al., 2004). On each trial, one of two pairs (AB and CD) of stimuli was presented and participants selected one member of the pair for possible reward. For AB pairs, choice of A(B) returned reward on 80%(20%) of trials; for CD pairs, choice of C(D) returned reward on 70%(30%) of trials (Frank et al., 2004). Based on participants’ choices while learning these pairings, we estimated separate learning rates for gains and losses (i.e., α_win_ and α_loss_) using a reinforcement learning (RL) model (Sutton and Barto, 1998). Learning rates, especially for gains, correlate positively with tonic levels of DA in the VS (Pessiglione et al., 2006), and were therefore considered a suitable means of assessing the impact of adversity on DA-mediated behavior.

Reward-based decision-making was assessed by means of the Delay Discounting task (DD) (Fishburn and Rubinstein, 1982, Hariri et al., 2006, van den Bos and McClure, 2013). On each of 88 separate trials, participants chose between a small immediate reward and a larger delayed reward. Across trials, the value of the immediate reward varied between $0 and $20 in increments of $1, and the delay was set to 7, 30, 90, or 180 days. The value of the delayed reward remained fixed at $20. Each unique combination of immediate reward value and delay duration was presented in random order. To model each participant’s choices, we first estimated indifference points for each delay using a logistic regression method, where an indifference point is defined as the point along the range of immediate reward values at a particular delay where the participant transitions from choosing the immediate reward to choosing the delayed reward. We then estimated how steeply participants discount the value of future rewards by fitting indifference points with a hyperbolic discounting function. Larger values of the discounting parameter reflect a preference for immediate over delayed rewards and have been associated with higher VS reactivity (Hariri et al., 2006).

## 2 Results study 1

**Fig. 1:**
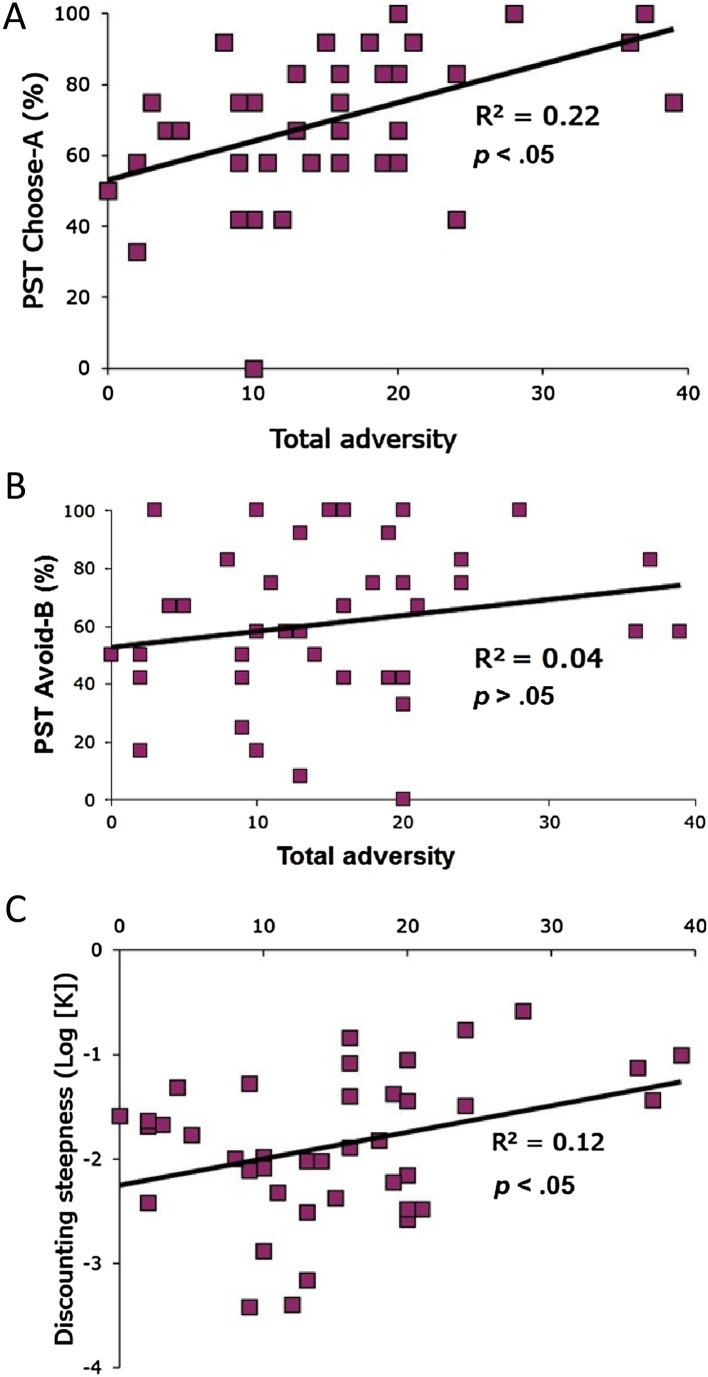
Associations Between Adversity and Reward-Based Learning and Decision-Making. (A) Adversity is positively correlated with reward-related learning r(38) = 0.547, p < 0.05(B) Adversity is not significantly correlated with loss-related learning r(38) = 0.189, p = 0.242 (C) Adversity is positively correlated with impulsive decision-making r(38) = 0.35, p < 0.05.

To test whether variation in normative exposure to adverse early-life events was associated with children’s reward-based learning, we correlated Total Adversity scores computed from the ELEQ with reward and loss-related learning from PST testing phase data. Adversity was positively correlated with reward-related learning, r(38) = 0.547, p = 0.002 (Fig. 1A), but not loss-related learning, r(38) = 0.189, p = 0.242 (Fig. 1B). To test whether variation in normative exposure to adverse early-life events was associated with children’s reward-based decision-making, we correlated Total Adversity scores computed from the ELEQ with delay discounting parameter estimates. Adversity was positively correlated with discounting parameter magnitude, r(38) = 0.35, p = 0.027, indicating that higher adversity scores were associated with more impulsive choice (Fig. 1C). Age was not related to any learning (reward-related r(38) = 0.097, p = 0.551; loss-related r(38) = −0.054, p = 0.741) or decision-making measures (delay-discounting r(38) = 0.143, p = 0.377).

Given evidence that variation in normative exposure to adverse early-life events was associated with variation in reward-based learning and decision-making, we were then interested in whether this association might be explainable, at least in part, in terms of variation in the brain’s response to the provision of small gains and losses. This was the goal of Study 2.

## 3 Methods study 2

### 3.1 Participants

Participants included 26 (12 females) children ranging between 9- and 12-years of age (M = 10.69, SD = 1.01). Twelve of the 26 children in the imaging protocol also participated in Study 1. Inclusion and exclusion criteria, consent and assent procedures, and compensation procedures were all identical to Study 1. All aspects of the study were conducted in accordance with the Declaration of Helsinki.

### 3.2 Measure of reward-based learning

To measure reward-based learning, participants were administered a simple reinforcement learning task presented as a game in which they could win points (Pessiglione et al., 2006). To increase children’s motivation to play, children were told that they would be given a monetary reward that scaled with the number of points earned. In fact, all children received the same monetary reward regardless of the number of points earned.

On each trial, participants were presented a pair of stimuli and chose one stimulus for possible gain (+10 points) or loss (-10 points). There were three types of stimulus pairs: gain pairs (AB), loss pairs (CD), and null (EF) pairs. For AB (or gain) pairs, choice of A returned 10(0) points on 80%(20%) of trials, and choice of B returned 10(0) points on 20%(80%) of trials. For CD (or loss) pairs, choice of C returned −10(0) points on 80%(20%) of trials and choice of D returned −10(0) points on 20%(80%) of trials. And for EF (or null) pairs, choice of either E or F returned 0 points.

Individual trials were 4000 ms in duration, and consisted of a 3000 ms stimulus presentation/response period and a 1000 ms feedback period. Individual trials were jittered in their presentation through use of an inter-trial interval that randomly varied in duration from 1 to 5 s in 1 s increments. During the stimulus/response period, one stimulus pair was presented, and participants selected one member of the pair by means of a button-press to an MRI-compatible button-box. Left button presses with the 2D finger were computer-coded as a choice of the stimulus presented on the left side of the screen; right button presses with the 3D finger computer-coded as a choice of the stimulus presented on the right side of the screen. Responses did not terminate the stimulus pair. Following the stimulus/response period, feedback appeared in the center of the screen for 1000 ms with either “+10”, “−10”, “0” or “Too Slow” if no response was registered in the preceding stimulus/response period. Position of the stimuli relative to fixation (i.e., left versus right) varied randomly from trial to trial. The order of gain-pair, loss-pair, and null trials was randomized for each participant.

Individual trials were administered in three 7-min runs. Each run consisted of 44 trials and included 20 AB trials, 20 CD trials, and 4 EF trials. All stimuli were motivationally neutral Snodgrass figures (Snodgrass and Vanderwart, 1980), with a unique set of 6 stimuli used for each of the three runs. The task was presented using a Lenovo laptop computer running E-Prime 2 software (Schneider et al., 2002).

### 3.3 MRI data acquisition

To mitigate fear and discomfort associated with the functional neuroimaging procedure, children were first exposed to MRI-like environment in the form of a mock scanner facility. MRI images were then acquired with a 3-Tesla Siemens Magnetom Prisma scanner and a Siemens Prisma 32-channel head coil. Functional T2*-weighted images were acquired with an ascending, interleaved slice order using a multiband echo-planar imaging (EPI) pulse sequence (TR = 686 ms; TE = 30 ms; FOV = 192 × 192 mm; flip angle = 54°; voxel size = 3 mm^3^, 64 × 64 matrix). We selected this sequence to maximize the sampling rate and permit better modeling of motion-related noise. A total of 3 runs of functional data were collected from each participant. Each functional run consisted of 650 volumes and lasted approximately 7 min.

After the completion of all 3 functional runs, we collected a high-resolution T1-weighted anatomical image using a 3D MPRAGE pulse sequence (192 slices; voxel size = 1 mm^3^, 256 × 256 matrix). The entire MRI procedure took approximately 1 hour to complete and participants were compensated with $10.00 cash and a $25.00 gift card.

### 3.4 fMRI data pre-processing

fMRI data were preprocessed using Statistical Parametric Mapping 12 (SPM12). For each run, volumes were spatially aligned to the first volume of the run that was acquired. Realignment parameters were archived and subsequently used to estimate participant motion during data acquisition. Participants with motion in excess of 3 mm of translation or 1.5 ° of rotation were dropped (18 participants were removed due to motion, and the imaging sample consisted of the remaining 26 participants). Motion parameters for each run were used as covariates of no interest in subsequent linear modeling. After coregistering functional and anatomical images, data were spatially normalized to Montreal Neurological Institute (MNI) space, and spatially smoothed via convolution with an 8 mm full-width at half-maximum Gaussian kernel.

### 3.5 Event-related modeling

Functional volumes were modeled by means of a general linear model (GLM) with separate predictors for six nuisance predictors (i.e., subject motion) and the following four events of interest:

• Wins: on gain-pair trials, instances when the participant gained 10 points;
• Misses: on gain-pair trials, instances when the participant gained 0 points;
• Losses: on loss-pair trials, instances when the participant lost 10 points;
• Avoids: on loss-pair trials, instances when participants lost 0 points.

Predictors for events of interest were created by convolving a vector of event onsets with a canonical hemodynamic response function (HRF), where, for all events, onsets were defined as the point in time that feedback was presented.

### 3.6 Reward-related region of interest (ROI) analysis

Reward-related Regions of Interest (ROIs) were defined through the use of the Neurosynth platform. A reverse-inference map of 671 studies that included the term “reward” in the abstract or introduction (http://www.neurosynth.org/; Yarkoni et al., 2011) was generated and the results of the meta-analysis were thresholded using a false discovery rate (FDR) correction for multiple comparisons of 0.01. We subsequently visualized the results in Mango. From this visualization, we identified and specified the coordinates of 4 ROIs (see Fig. 4A/B), including the left and right ventral striatum (VS), and the left and right ventromedial prefrontal cortex (vmPFC). We then used the MarsBar Region of Interest toolbox for SPM to extract and average beta coefficients for all voxels within each of the four ROI’s, separately for each of the four predictors (i.e., Wins, Misses, Losses, and Avoids − see Section 4.5). These coefficients were then used to compute selected contrasts and brain-behavior correlations. Following Pessiglione et al. (2006), gain-related activity was computed as the contrast of Wins − Misses corrected for multiple comparisons at p < 0.05 via Bonferroni’s procedure, and loss-related activity as the contrast of Losses − Avoids corrected for multiple comparisons at p < 0.05 via Bonferroni’s procedure. To examine associations of gain- and loss-related activity to behavior and early adversity, statistically significant contrasts of Wins − Misses or Losses − Avoids were correlated with learning rates to wins and losses and Total Adversity scores respectively.

## 4 Results study 2

### 4.1 Behavioral results

**Fig. 2:**
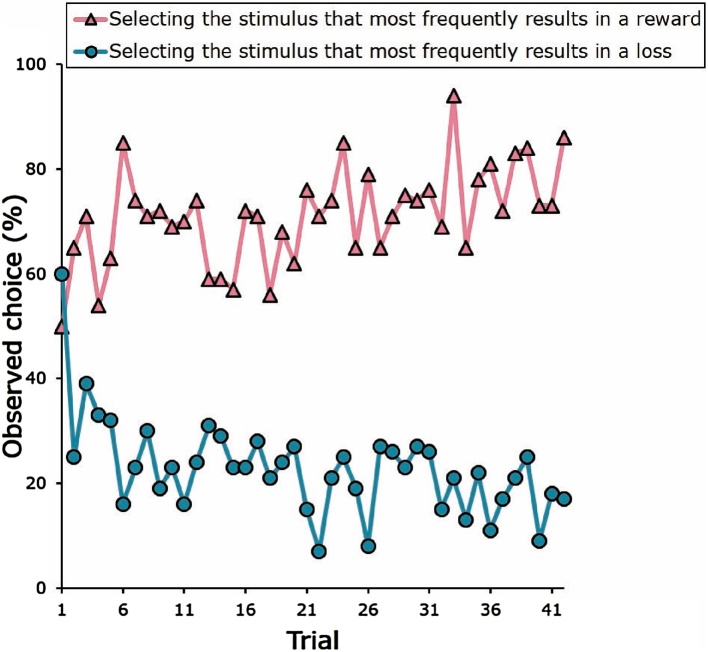
Observed choice to gain-pair and loss-pair stimuli. Over the course of the trials, participants learned to select the stimulus that more frequently results in a reward (pink triangles) and avoid the stimulus that more frequently results in a loss (blue circles). (For interpretation of the references to colour in this figure legend, the reader is referred to the web version of this article.)

Over time, children learned to select A on AB trials (Fig. 2, pink triangles) and avoid C on CD trials (Fig. 2, blue circles), indicating that they learned to select stimuli that maximized gains and minimized losses. Gain-pair accuracy, defined as the proportion of AB trials of which participants chose A, was uncorrelated with loss-pair accuracy, defined as the proportion of CD trials of which participants chose D (r(24) = 0.334, p = 0.095). Neither gain-pair accuracy (r(24) = 0.075, p = 0.716) nor loss-pair accuracy (r(24) = 0.040, p = 0.846) were associated with age.

**Fig. 3:**
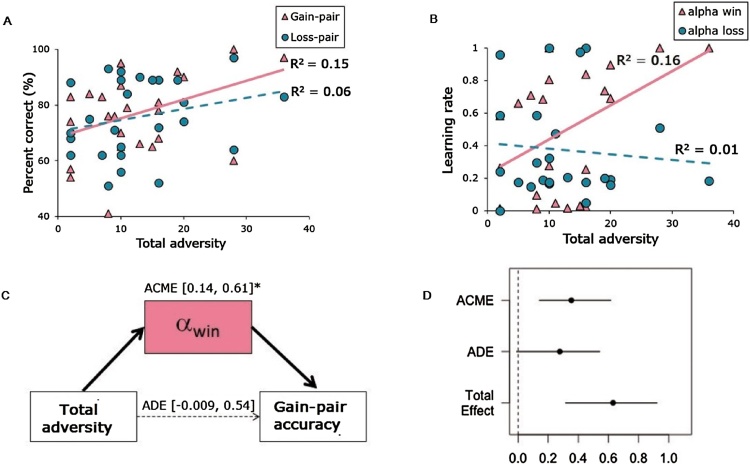
Associations Between Adversity And Reward-Related and Loss-Related Learning. (A) Adversity was positively correlated with gain-pair accuracy (pink triangles) r(24) = 0.39, p < 0.05, but not loss-pair accuracy, r(24) = 0.25, p =0.218 (blue circles). (B) Adversity was positively correlated with learning rate to gains (pink triangles) r(24) = 0.40, p < 0.05, but not learning rate to losses r(24) = 0.1, p = 0.627 (blue circles). (C and D) The association between adversity and gain-pair accuracy was fully mediated by learning rate to gains. ACME = Average causal mediation effect; ADE = Average direct effect. (For interpretation of the references to colour in this figure legend, the reader is referred to the web version of this article.)

To test for possible associations between learning and adversity, we first correlated gain- and loss-pair accuracy with Total Adversity scores. Total Adversity was positively associated with gain-pair, r(24) = 0.39, p = 0.048, but not loss-pair accuracy, r(24) = 0.25, p = 0.218 (Fig. 3A). Then, to specifically examine whether dopamine-mediated aspects of reward-based learning explained the association between adversity and choice behavior, we modeled choice behavior using an RL model, with separate learning rates for gains (α_win_) and losses (α_loss_). Consistent with findings from the PST observed in Study 1, adversity was associated with learning rates for gains, r(24) = 0.40, p = 0.042, but was not associated with learning rates for losses r(24) = 0.1, p = 0.627 (Fig. 3B). Importantly, the association between adversity and choice behavior was fully mediated by learning rate to gains (Fig. 3C and D).

### 4.2 ROI activation, relation to behavior

**Fig. 4:**
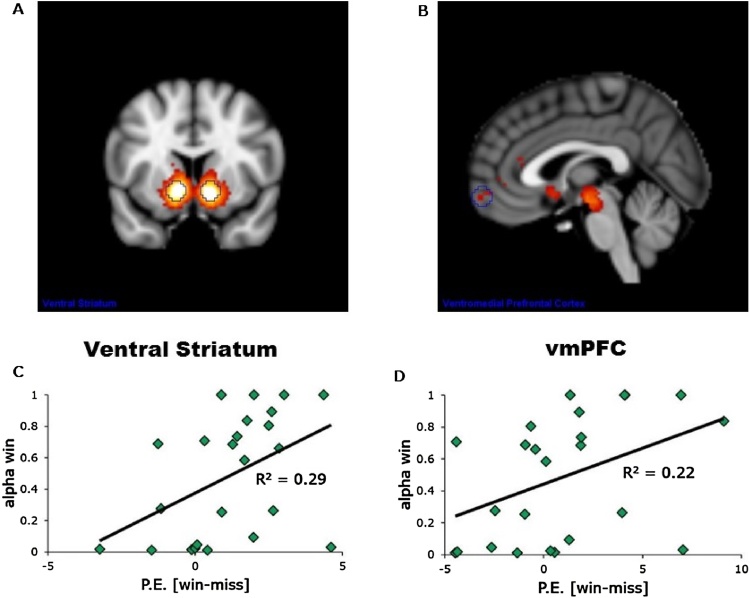
Neuroimaging Results. (A and B) Neurosynth meta-analysis of 671 studies that included the word “reward”. Both the VS and vmPFC are preferentially related to the term “reward”. Based on the meta-analysis ROIs were created for both the VS [-12,10,-9; 12,10,-9] as well as the vmPFC [2,62,−10; −2,62,−10], visible in blue. (C and D) Reward-relate activity correlated positively with learning rate to gains in the VS r(24) = 0.54, p < 0.05, and vmPFC r(24) = 0.47, p < 0.05.

Both left VS and left vmPFC showed significant gain- but not loss-related activation. Gain-related activity predicted learning rates to gains (Fig. 4C/D) both for the left VS, r(24) = 0.54, p < 0.05, and the left vmPFC, r(24) = 0.47, p < 0.05.

### 4.3 Adversity-behavior association partially explained by VS activation

**Fig. 5:**
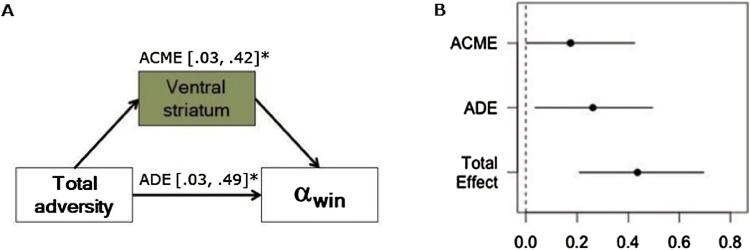
Mediation Analysis. (A and B) Reward-related activity in the VS partially mediated the relationship between adversity and learning rate to gains. ACME = Average causal mediation effect; ADE = Average direct effect.

To examine whether the association between adversity and reward-related learning could be explained, at least in part, by reward-related activity in our ROIs, we tested separate mediation models for the VS and vmPFC. Reward-related activity in the left VS partially mediated the association between adversity and learning rate for gains (Fig. 5A and B). No other mediation models were significant.

## 5 Discussion

Individual differences in children’s reward-based learning and decision-making predict many important physical and psychological outcomes. The results of the present study are important as they contribute to an understanding of the origin of these individual differences.

First, we found that early-life adversity predicts differences in typically developing children’s reward-based learning and decision-making. Adversity experienced early in development has been linked to increased impulsivity and reward incentive salience, both in animals and in humans (Chugani et al., 2001, Hall, 1998). In rats, maternal-separation and isolate-rearing increase impulsivity and hyperactivity, with effects more pronounced in measures of impulsive action than impulsive choice (Lovic et al., 2011). In humans, exposure to adversity early in life is associated with heightened ADHD symptomology including greater impulsivity and hyperactivity (Laucht et al., 2007). In the current study, we found that children who had experienced more frequent and intense adverse events early in life discounted temporally displaced rewards more steeply and showed potentiated reward-based learning as compared to children who had experienced less frequent and intense adverse early life events. Interestingly, the association between adversity and learning was specific to aspects of approach learning, including the likelihood of selecting previously rewarded stimuli and RL-model estimates of learning rate to gains. There were no corresponding associations between adversity and avoidance learning.

Second, we found that the link between adversity and reward-processing could be explained, at least in part, by differences in ventral striatal response to rewards. Consistent with earlier studies (Pessiglione et al., 2006), rewards were associated with activity in the VS and the vmPFC, and reward-related activity in these regions predicted reward-related learning, including RL-model estimates of learning rate to gains. Interestingly, of these two regions, it was the VS that partially mediated the association between adversity and learning rate to gains. Taken together then, our findings point to a link between adversity, VS physiology, and reward-related behavior.

One plausible explanation for our findings is that early adversity contributes to hyper-dopaminergic functioning in the VS. Indeed, pharmacologically induced changes in striatal DA impact VS activity and reward-based learning in ways that are similar to variations in adversity; an increase in striatal DA induced by the anti-Parkinsonian medication L-DOPA for example, increases learning rates and VS response to gains but has no effect on learning and striatal response to losses (Pessiglione et al., 2006). The idea that early adversity contributes to hyper-dopaminergic functioning in the VS is certainly consistent with evidence from animal studies and recent human imaging studies (Egerton et al., 2016, Oswald et al., 2014). In rodents, for example, early adversity has been linked to increases in tonic and phasic DA, as reflected in measures of basal DA (Abercrombie et al., 1989, Hall et al., 1998) and evoked response to amphetamine administration (Piazza and Le Moal 1996) respectively. Similarly in humans, adversity experienced in childhood has been associated with elevated levels of dopamine in adulthood (Egerton et al., 2016), and increased ventral striatal dopamine response to amphetamine. In other cases however, there appears to be a blunted sensitivity to rewards as measured by fMRI (Boecker et al., 2014, Dillon et al., 2009, Hanson et al., 2016, Mehta et al., 2010, Weller and Fisher, 2012), which may be indicative of hypo-dopaminergic striatal functioning; these contradictory findings may be a result of differences in the timing, type, and severity of adversity experienced.

Such discrepancies highlight several critical limitations of the present study. One limitation concerns the fact that we used the ELEQ to measure children’s exposure to adversity. The use of the ELEQ reflects our interest in normative variation in adversity; however, this measure has not been widely used and awaits proper validation against other more standardized measures such as ACES. A second limitation is that we did not collect information about the pubertal status of our participants. Adolescence is a developmental period during which individuals exhibit a hypersensitivity to rewards (Van Leijenhorst et al., 2010), and therefore a completely unblemished picture of reward processing in late childhood would remove any possible influence of pubertal onset. That said, none of our measures of reward-based learning and decision-making were correlated with age, suggesting that the current findings do not simply document variation related to maturation.

The findings have important implications for our understanding of human development by providing evidence that the functional calibration of the reward system is sensitive to variation in adversity that falls within the normative range. Early childhood is considered a sensitive period as variations in the quality of experience during this time have lasting implications for behavioral, neurophysiological, and neuroendocrine organization. This may be especially true of the reward system, given its role in learning and motivated behavior specifically, and its importance for adaptation to different environments more generally (for discussion see Gatzke-Kopp, 2011, Frankenhuis and Weerth, 2013). For example, using tasks that engaged the reward system, our results show that children who experienced greater adversity exhibited potentiated reward-related learning and impulsive decision-making. Importantly, the adverse life events did not fall outside the normative range; thus, these results highlight the sensitivity of the reward system to indicators of environmental quality.

Our findings have potentially important health and policy implications as well. Early variation in dopamine-mediated traits, such as reward incentive salience, impulsivity, and striatal reactivity to rewards, have been linked to increased lifetime risk of substance use/addiction, relationship problems, poor financial decision-making, and low educational achievement (Moffitt et al., 2011). A more detailed understanding of the impact of adversity on the developing brain may ultimately help to alleviate some of the financial burden and human suffering related to these psychosocial issues.

Of course, much remains to be learned about the impact of early adversity on neurocognitive development and reward processing. It is unclear whether mechanisms relating adversity and reward processing are molecular (e.g., D2-receptor down-regulation; Hall et al., 1998), anatomical (e.g., volumetric changes within the reward system or in the integrity of fiber tracts connecting the reward system to other brain regions, etc.), or involve interactions with other systems such as the stress system (see Gatzke-Kopp, 2011, Piazza et al., 1991, Piazza and Le Moal, 1996, Pruessner et al., 2004). These questions represent critical avenues for future research.

## Conflict of Interest

None.
