# Supplementary material for: Ventral striatal activity links adversity and reward processing in children
Source: Dev Cogn Neurosci. 2017 Apr 15;26:20–7. doi: 10.1016/j.dcn.2017.04.002 (PMC6987763; doi:10.1016/j.dcn.2017.04.002)
Supplement: Supplementary file 1 [file mmc1.docx]

**Supplementary Materials**

***Delay Discounting***

Because children exhibit choice “jitter” around indifference points, we developed an objective means of identifying indifference points based on logistic regression and the fitting of a logistic sigmoid function to choice data. For each delay period, the choice of delayed vs. immediate reward was defined categorically as 0 and 1, respectively. All choices at a particular delay, $x^{\left( i \right)}$, were then fit with the generalized logistic sigmoid function, $h_{\theta}$, modified by fitted coefficients $\theta$ (Equation 1).

Equation 1

$$h_{\theta}\left( x^{\left( i \right)} \right)=\frac{1}{1+e^{-\theta^{T}x^{\left( i \right)}}}$$

The values, $\theta^{T}$, were determined through the minimization of a regularized cost function that reflects the degree of fit between the logistic function and the participant data (Equation 2).

Equation 2

$$J\left( \theta\right)=-\frac{1}{m}\sum_{i=1}^{m} \left[ y^{(i)}\log\left( h_{\theta}\left( x^{\left( i \right)} \right) \right)+\left( 1-y^{\left( i \right)} \right)\log\left( 1-h_{\theta}\left( x^{\left( i \right)} \right) \right) \right]+\lambda\frac{1}{2}\sum_{j=1}^{n} \theta_{j}^{2}$$

Because the logistic function can be interpreted as indicating the probability of selecting an immediate reward of a given value, an indifference point at a particular delay was defined as the point at which the probability of selecting the immediate reward was equal to 0.5. Having determined indifference points in this way, each participant’s four indifference points were modeled by means of a hyperbolic function (Equation 3; Reed et al. 2012), where V is the subjective value of the larger delayed reward amount (A) at a given delay (D). The parameter *k* denotes the discounting rate observed (i.e., the steepness of the curve or how fast the subjective value drops as a function of the delay).

Equation 3

$$V=\frac{A}{1+(kD)}$$

***Computational Modelling***

To assess subcomponents of feedback-based learning, a reinforcement learning (RL) model was fit to each participant’s behavioral data (Sutton & Barto, 1998). These models have been successfully applied to describe the behavior of teenagers and children (van den Bos & McClure, 2013). The RL model uses the prediction error (δ) to update the decisions weights (*w*) associated with each choice option (e.g., *A, B, C, or D* in experiment 1). The prediction error compares the actual outcome with the expected outcome. Whenever feedback is better (worse) than expected, the model will generate a positive (negative) prediction error, which is used to increase (decrease) the weight of the decision associated with the chosen option (e.g., option *A*). The impact of the prediction error on the updating is scaled by the learning parameter alpha (0<α<1). Based on previous research, we assumed that gains and losses are asymmetrically updated (Kahnt et al., 2009; van den Bos & McClure, 2013), thus we modeled the behavior with two independent learning rates; (α_pos_) for positive feedback and (α_neg_) for negative feedback.

To model trial-by-trial choices, we used the soft-max choice rule to compute the probability (*P*) of choosing the most rewarding target of a presented pair of choices (e.g. probability of choosing A of the AB pair experiment 1) on trial (*t*) as the difference in the decision weights in each trial (w_t_) associated with each option, passed through a sigmoid function (Montague, Hyman, & Cohen, 2004). For example, when stimulus pair *AB* is presented, the probability of choosing *A* is determined by:

Equation 4

$${P\left( A \right)}_{t}= \frac{1}{1+e^{-\beta\cdot(w \left( A \right)_{t}-w \left( B \right)_{t})}}$$

where the β parameter is a free parameter that indicates the sensitivity of the subject to the differences in decision-weights. The lower the β parameter, the more random the choices appear. After each decision, the prediction error (δ) is calculated as the difference between the outcome received (r = 1 for positive feedback and 0 for negative feedback) and the decision weight (*w*_t_) for the chosen stimulus:

**Equation 5**

$\delta_{t}=r_{t}-w(chosen\_stimulus)$

Subsequently, the decision weights are updated according to:

Equation 6

$w_{t+1}=w_{t}+ \lambda\times\alpha_{outcome}\times\delta_{t}$

where $\lambda$ is 1 for the chosen and 0 for the unchosen stimulus, $\alpha$_outcome_ is a set of learning rates for positive (α_pos_) and negative feedback (α_neg_), which scale the effect of the prediction error on the future decision weights and thus subsequent decisions. For example, a high learning rate for positive feedback but a low learning rate for negative feedback indicates that positive feedback has a high impact on future behavior, whereas negative feedback will hardly change future behavior. Importantly, at the beginning of the experiment the decision weights (*w*) for each option was always set to the same value (0.5 for the PST, and 0 for the Instrumental Learning Task, in both cases this is the mean of the expected outcomes), indicating that at the beginning of the experiment the participants had the same expectation of rewards for each option. The learning rates and sensitivity parameter were individually estimated by fitting the model predictions to participants’ actual decisions. Finally, note that for the Instrumental Learning Task, we fit gain and loss trials separately given that the zero outcome in the gain and loss context has a different meaning. That is, in the gain context the zero outcome is seen as a missed gain and in the loss context it means avoiding a loss. Thus our modeling is based on the (uncontroversial) assumption that a missed gain is not the same thing as an actual loss, nor is avoiding a loss the same as gaining a point. As a result, we labeled the learning rates as follows; α_gain_ and α_miss_ in the gain context and α_loss_ and α_avoid_ in the loss context.

We used a robust combination of grid-search and maximum likelihood estimation using the Nelder–Mead simplex algorithm implemented in the optim function in R to estimate the model parameters for each participant. Each point on the grid served as a starting position for the minimization function, which is then used to find the parameters that maximize the log likelihood for an individual. The grid point that produced the maximum over all starting positions was selected as starting point for finding the final solution.

For quality assessment, the dual learning rate model was compared to several other models; random choice, single learning rate and win-stay lose-shift (WSLS). The single learning rate is just as described above, but with a single learning rate for both type of outcomes. The random choice model would choose each option with 50% probability (no learning). Finally, the WSLS also suggests people use a simple choice rule without any learning.

For model selection purposes we computed the Bayesian information criterion (BIC), where lower BIC values indicate better fit. These model comparisons indicate that, on the whole, the dual learning rate model best described the behavior on both experiment 1 (PST) and 2 (Instrumental Learning Task) (see Table S1). Finally, if subject level fits of the dual learning rate model were not significantly different from the non-learning models these subjects were excluded from further analyses of parameter estimates; parameters of the learning model could not be reliably estimated in the absence of learning. This led to the exclusions of 12 subjects in experiment from the PST dataset, and 1 in the Instrumental Learning dataset.

Finally, results for the Kolmogorov-Smirnov test indicated that the distributions of all of the model parameters deviate significantly from a normal distribution (all *p’s* < .001), so for subsequent analyses all parameters were first log transformed and then scaled.

Tables

Table S1.

**Model comparisons for the Probabilistic Selection Task and the Instrumental Learning Task.**

|  |  | single alpha | dual alpha | WSLS |
| --- | --- | --- | --- | --- |
| PST | BIC | 4378 | 4292* | 18687 |
| Instrumental Learning Task –gain | BIC | 1832 | 1824* | 25035 |
| Instrumental Learning Task –loss | BIC | 1854 | 1846* | 26048 |
